# Characterization of a new high copy *Stowaway* family MITE, *BRAMI*-1 in *Brassica* genome

**DOI:** 10.1186/1471-2229-13-56

**Published:** 2013-04-02

**Authors:** Perumal Sampath, Sang-Choon Lee, Jonghoon Lee, Nur Kholilatul Izzah, Beom-Soon Choi, Mina Jin, Beom-Seok Park, Tae-Jin Yang

**Affiliations:** 1 Dept. of Plant Science, Plant Genomics and Breeding Institute, and Research Institute for Agriculture and Life Sciences, College of Agriculture and Life Sciences, Seoul National University, Seoul, 151-921, Republic of Korea; 2 National Instrumentation Center for Environmental Management, College of Agriculture and Life Sciences, Seoul National University, Seoul, 151-921, Republic of Korea; 3 National Academy of Agricultural Science, Rural Development Administration, 150 Suinro, Suwon, 441-707, Republic of Korea

**Keywords:** Miniature Inverted-repeat Transposable Element (MITE), MITE insertion polymorphism (MIP), *Brassica* species, Evolution, *BRAMI*-1

## Abstract

**Background:**

Miniature inverted-repeat transposable elements (MITEs) are expected to play important roles in evolution of genes and genome in plants, especially in the highly duplicated plant genomes. Various MITE families and their roles in plants have been characterized. However, there have been fewer studies of MITE families and their potential roles in evolution of the recently triplicated *Brassica* genome.

**Results:**

We identified a new MITE family, *BRAMI*-1*,* belonging to the *Stowaway* super-family in the *Brassica* genome. *In silico* mapping revealed that 697 members are dispersed throughout the euchromatic regions of the *B. rapa* pseudo-chromosomes. Among them, 548 members (78.6%) are located in gene-rich regions, less than 3 kb from genes. In addition, we identified 516 and 15 members in the 470 Mb and 15 Mb genomic shotgun sequences currently available for *B. oleracea* and *B. napus*, respectively. The resulting estimated copy numbers for the entire genomes were 1440, 1464 and 2490 in *B. rapa, B. oleracea* and *B. napus,* respectively. Concurrently, only 70 members of the related *Arabidopsis ATTIRTA*-1 MITE family were identified in the *Arabidopsis* genome. Phylogenetic analysis revealed that *BRAMI*-1 elements proliferated in the *Brassica* genus after divergence from the *Arabidopsis* lineage. MITE insertion polymorphism (MIP) was inspected for 50 *BRAMI*-1 members, revealing high levels of insertion polymorphism between and within species of *Brassica* that clarify *BRAMI*-1 activation periods up to the present. Comparative analysis of the 71 genes harbouring the *BRAMI*-1 elements with their non-insertion paralogs (NIPs) showed that the *BRAMI*-1 insertions mainly reside in non-coding sequences and that the expression levels of genes with the elements differ from those of their NIPs.

**Conclusion:**

A *Stowaway* family MITE, named as *BRAMI*-1, was gradually amplified and remained present in over than 1400 copies in each of three *Brassica* species. Overall, 78% of the members were identified in gene-rich regions, and it is assumed that they may contribute to the evolution of duplicated genes in the highly duplicated *Brassica* genome. The resulting MIPs can serve as a good source of DNA markers for *Brassica* crops because the insertions are highly dispersed in the gene-rich euchromatin region and are polymorphic between or within species.

## Background

The large-scale sequencing of eukaryotic genomes has revealed that transposable elements (TEs) are present ubiquitously and occupy large fractions of genomes: 5% in yeast, 35% in rice, 45% in human, and up to 85% in maize [1-9]. TEs are classified into two classes based on their transposition mechanism. Class I mobile genetic elements, or retrotransposons, are replicated through RNA intermediates by a copy-and-paste mechanism, whereas Class II mobile genetic elements, or DNA transposons, move directly from DNA via a cut-and-paste mechanism [1,2,10].

Miniature inverted-repeat transposable elements (MITEs) are Class II DNA transposons that are non-autonomous, with defective or absent of coding genes. MITEs were identified in the maize genome [11] and later found in *Arabidopsis*, rice, grape, mosquito, fish, bacteria and human as well as in several other genomes [1,12-15]. Due to their extremely high copy numbers, MITEs can account for a significant fraction of a eukaryotic genome (i.e. >8% of the rice genome) even though the size of element itself is small [16]. Individual MITEs are usually less than 600 bp and A/T rich, with terminal inverted repeats (TIRs) and 2–11 bp target site duplication (TSD) sequences [1,10]. MITEs, which are relatively stable in the genome, are often closely associated with genic regions and thus can affect gene expression patterns [16,17]. Some MITEs are involved in up-regulation of host genes by providing additional recognition sequences or polyA signals to host genes [14,18,19]. MITE insertion into regulatory regions may cause disruption or promotion of gene expression [18]. Recent studies have found that MITEs are also a source of small interfering RNA (siRNA) evolution and may play an important role in gene regulation and epigenetic mechanisms [16,20-22]. MITE transposition into a new region of the genome causes insertion polymorphisms among accessions of same species that can be useful tools for development of various markers [23,24].

The *Brassicaceae* family includes 338 genera and 3700 species, which serve as sources of vegetable, fodder, condiments and oil, with wide range of morphologies, such as Chinese cabbage, mustard, cabbage, broccoli, oilseed rape, and other leafy vegetables. The model plant, *Arabidopsis thaliana* is a close relative of the *Brassica* species and belongs to the same family*.* As a model *Brassica* crop, the *B. rapa* genome sequence spanning 256 Mb euchromatin chromosome spaces was completed recently and released to the public [25].

Comparative analysis of *Brassica* species with *A. thaliana* has revealed up to two additional rounds of recent genome duplication: one triplication and one allopolyploidization that is the major factor responsible for the increased genome size of *Brassica*[25-27]. In addition, TEs also contribute to increase the genome size of the *Brassica* species and to genome evolution [28]. The completed genome sequence of *B. rapa* revealed that at least 39.5% of the genome contains TEs [25].

In this study, we identified a new MITE named *Brassica rapa*MITE (*BRAMI)*-1, which is present in more than 1400 copies in the genome of each of three *Brassica* species. We inspected its characteristics and distribution and inferred its potential involvement in the evolution of duplicated genes in the highly replicated *Brassica* genome. We also discovered high amounts of insertion polymorphism inter- and intra-species, which can serve a good source of genetic markers in the *Brassica* species.

## Results

### Characterization of *BRAMI*-1 in *Brassica*

**Figure 1:**
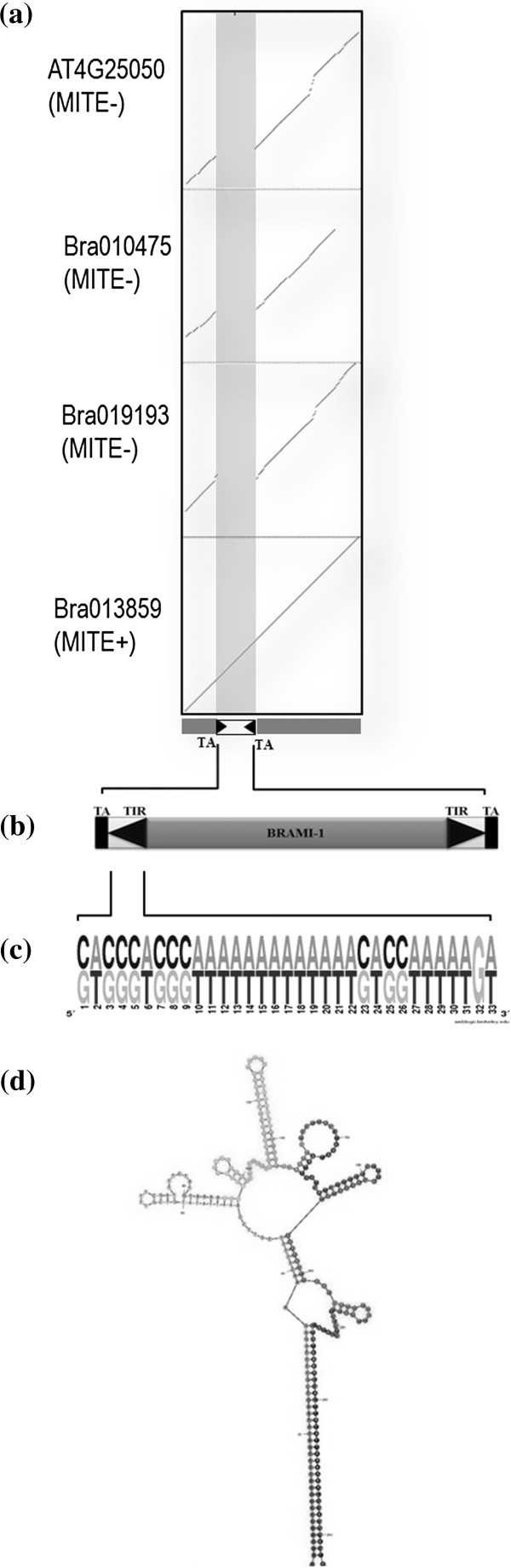
**Identification and characterization of the *BRAMI-*1 elements.** (**a**) Dotplot analysis of Bra013859 and the related empty sites in its two non-insertion paralog (NIP) genes, Bra019193 and Bra010475 from *B. rapa* and its orthologue At4g25050 from *A. thaliana* (**b**) The structure of *BRAMI-*1 showing its characteristic properties, TA Target site duplication (**c**) Conserved 33 bp TIR sequences shown by Weblogo analysis (**d**) Hypothetical secondary structure and expected loop formation predicted by mfold.

We identified a 260 bp MITE in the *Brassica rapa* BAC clone, KBrB059A03 using MUST, a *de novo* program for MITE identification, and additional manual inspection. MITE characterization on *B. rapa* contig (KBrB059A03) using MUST yielded 291 candidate MITEs and further careful manual inspection of each candidate MITE for TIR and TSD using self-BLAST (http://blast.ncbi.nlm.nih.gov/) led to the identification of *BRAMI-1*. Comparison of *BRAMI−1* against the repeat database (http://www.girinst.org/) showed 77% similarity to a reported *Stowaway* MITE, *ATTIRTA*-1 in *A. thaliana*[29]. Perfect MITE insertion was confirmed by comparing one of the representative *B. rapa* genes (Bra013859) harboring a *BRAMI*-1 insertion with the related empty sites in its non-insertion paralogs (NIPs) (Bra010475 and Bra019193) from *B. rapa* syntenic blocks and its ortholog (At4g25050) in *A. thaliana* (Figure 1a, b). The MITE included 33 bp of highly conserved A/T rich (>69%) TIRs and was flanked with a unique di-nucleotide TA target site duplication (TSD), which are distinct characteristics of the *Stowaway* super-family MITEs (Figure 1b, c). The secondary structure of the MITE was predicted using mfold (Figure 1d), which showed a potential DNA hairpin-like secondary structure.

**Figure 2:**
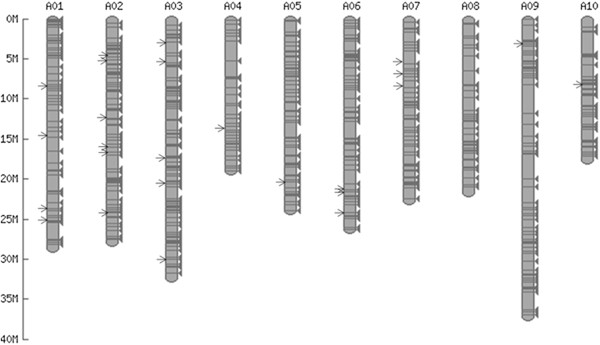
***In silico *mapping of *BRAMI-*1 elements in 256 Mb of *B. rapa *pseudo-chromosomes.** Arrows indicate the positions of the 25 members used for MIP analysis. The exact physical positions of the 697 *BRAMI-*1 members are listed in Additional file 1.

BLASTn searches revealed a total of 697 *BRAMI*-1 elements in the 256 Mb *B. rapa* genome sequence. *In silico* mapping of these elements on the *B. rapa* pseudo-chromosomes showed that they were evenly distributed in the euchromatin regions of the *B. rapa* genome (Figure 2). The physical positions of the 697 *BRAMI*-1 elements in the *B. rapa* genome are listed in Additional file 1. On average, 70 *BRAMI*-1 elements were found on each pseudo-chromosome. MITE density analysis (chromosome size/no. of MITEs per chromosome) shows chromosome 3 (31.72 Mb), which is the second largest in size, has the high MITE density (MITE/0.28 Mb), while the largest chromosome 9 (37.12 Mb) had the less MITE density (MITE/0.44 Mb).

We found 516 and 15 copies in 470 Mb of *B. oleracea* and 15 Mb of *B. napus* shotgun sequences, respectively. Based on this, the total numbers of the *BRAMI*-1 MITE members were estimated as 1440, 1464 and 2490 in the whole genomes of *B. rapa, B. oleracea* and *B. napus,* respectively (Table 1). By contrast, in *A. thaliana* we found only 70 copies of *ATTIRTA*-1, the closest *Arabidopsis* relative of *BRAMI-*1. Simple comparison revealed that the copy numbers of these MITEs in *Brassica* genomes are 20–35 times more than that of *Arabidopsis*.

**Table 1:** **Summary of observed and predicted copy numbers of the *BRAMI-*1 elements in *Brassica *relatives**

**MITE**	***BRAMI*-1**			***ATTIRTA*-1**
	***B. oleracea***	***B. rapa***	***B. napus***	***A. thaliana***
Database type	GSS	Pseudo-chromosomes	GSS	Whole genome
Database size	470 Mb	256 Mb	15 Mb	119 Mb
Total copies	399	697	11	70
(>80% similarity)	123	401	4	34
Average length of the GSS sequence	700 bp	N/A	700 bp	N/A
Estimated Genome Size [30]	696 Mb	529 Mb	1132 Mb	157 Mb
Estimated copies in the whole genome	1464	1440	2490	44

BLASTn was performed at the local database (http://imcrop.snu.ac.kr).

N/A: not applicable.

### Phylogenetic analysis of the *BRAMI*-1 elements

**Figure 3:**
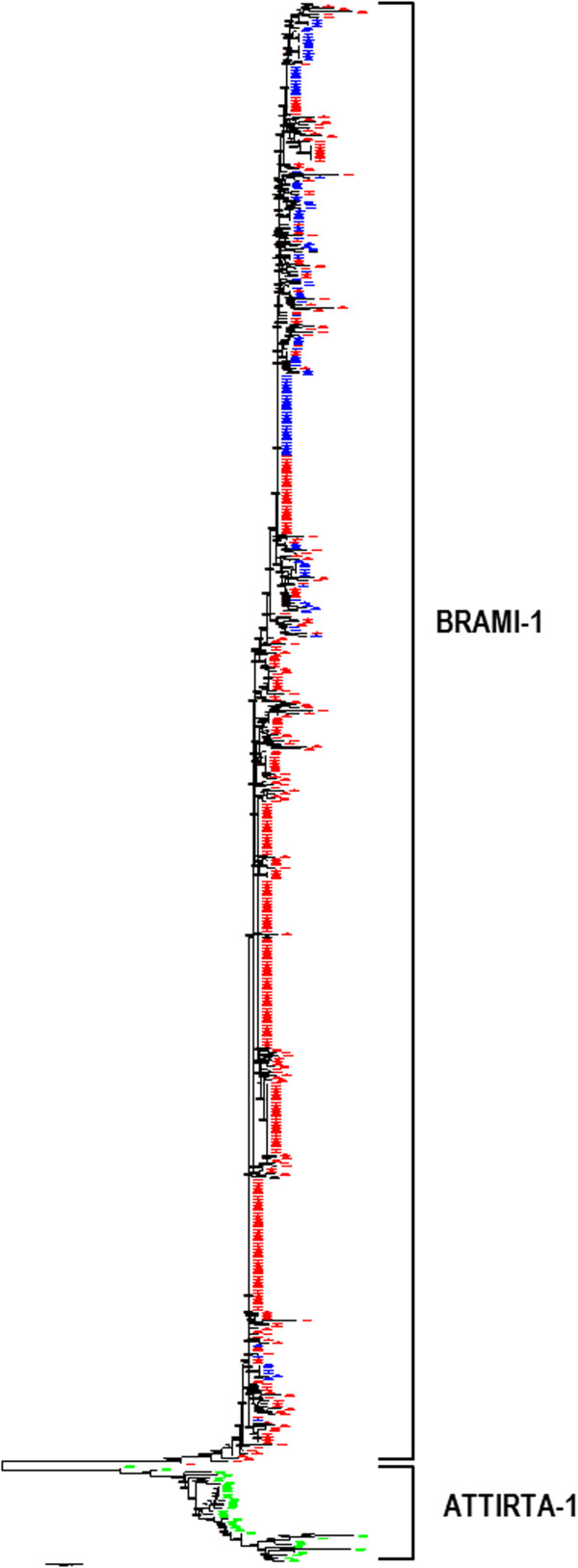
**Phylogenetic tree of *BRAMI*-1 elements from *Brassica *species and *ATTIRTA*-1 from*A. thaliana.*** Relatively intact MITE members showing 80% similarity to the characteristic MITE structure were used for the analysis. A total of 528 *BRAMI*-1 members including 401, 123, and 4 from *B. rapa* (red), *B. oleracea* (blue), and *B. napus* (black), respectively, and 34 *ATTIRTA*-1 members (green) were compared. Sequence alignment was conducted using ClustalW and then the phylogenetic tree was generated using the neighbor joining method with 500 bootstrap replicates.

Phylogenetic analysis was conducted for 528 nearly intact MITE members that have >80% similarity to *BRAMI-*1: 401 members from *B. rapa*, 123 from *B. oleracea*, and four from *B. napus*. In addition, 34 *ATTIRTA-*1 members from *A. thaliana* were included. The *ATTIRTA-*1 members formed a separate clade from the *Brassica* members, and they were very diverse among themselves. By contrast, *BRAMI*-1 members from the three *Brassica* species were highly conserved and were interspersed with each other (Figure 3) indicating they were rapidly amplified in the *Brassica* genome after divergence from *Arabidopsis*. Due to their high sequence similarity, we could not distinguish any separate clades for the *BRAMI*-1 family members in the *Brassica* species.

### *BRAMI*-1 insertion in genic regions of the *B. rapa* genome

We inspected the insertion sites of the 697 *BRAMI*-1 elements in the *B. rapa* genome using the annotated *B. rapa* genome database [31]. The analysis showed that 548 members (78.6%) were located in gene-rich regions, less than 3 kb from genes. Among them, 71 (10.2%) were inside the gene structure, specifically in introns, and 281 (40.3%) were within less than 1 kb of a gene (Table 2).

**Table 2:** **Summary of the insertion positions of 697 *BRAMI-*1 elements in the *B. rapa *genome**

**Insertion position**	**Number of elements**	**Percentage of elements**
Gene	71	10.2
Near Genic Regions (<1 kb)^a^	281	40.3
Near Genic Regions (1 kb to <2 kb)^a^	134	19.2
Near Genic Regions (2 kb to <3 kb)^a^	62	8.9
Intergenic Region (>3 kb)^a^	149	21.4
Total	697	100.0

^a^ Distance from nearest gene.

We closely inspected the 71 genic insertions by comparing with their NIPs from triplicated chromosomal blocks. Similar numbers of insertions were identified in tri-, di-, and mono-copy genes (20, 26, and 24 insertions, respectively; Table 3) indicating that multi-copy genes did not preferentially contain *BRAMI-*1 insertions. Comparison of genes containing the *BRAMI-*1 insertion and their NIPs genes in the triplicated blocks revealed that all of the elements resided in intronic regions.

**Table 3:** **Insertion positions and names of the 71 genes harboring *BRAMI*-1 elements in intronic regions and list of their orthologous genes in *Arabidopsis *and NIPs in the triplicated blocks of the *B. rapa *genome**

	**MITE No**	**Chr No**	**MITE start**	**MITE end**	**Ortholog from *A. thaliana***	**Triplicated blocks in*B. rapa***^**z**^		
						**LF**	**MF1**	**MF2**
THREE COPY GENES	23	A01	8110084	8109821	At4g25050	**Bra013859**	Bra019193	Bra010475
	83	A02	4177719	4177456	At5g17300	Bra008563	Bra006394	**Bra023610**
	176	A03	5778627	5778365	At5g55050	Bra002937	Bra035549	**Bra028994**
	188	A03	8910390	8910639	At2g37940	Bra005148	Bra017148	**Bra000029**
	219	A03	17370042	17370087	At3g15820	Bra027205	Bra021138	**Bra001607**
	220	A03	17370087	17370331	At3g15820	Bra027205	Bra021138	**Bra001607**
	299	A04	13405505	13405756	At2g30110	Bra018338	**Bra021611**	Bra022779
	303	A04	14136797	14136887	At2g31500	Bra018236	**Bra021727**	Bra022844
	346	A05	7674854	7674713	At2g29980	**Bra018348**	Bra021599	Bra022767
	347	A05	7674974	7675006	At2g29980	**Bra018348**	Bra021599	Bra022767
	349	A05	8733844	8733992	At4g04640	Bra029511	Bra000802	**Bra018503**
	368	A05	16917820	16918070	At3g20770	**Bra035746**	Bra023927	Bra001802
	425	A06	14973843	14973752	At5g64740	**Bra024324**	Bra037793	Bra031904
	443	A06	22195786	22196049	At3g28050	**Bra025321**	Bra033037	Bra039062
	450	A06	24123977	24123793	At2g01430	**Bra024888**	Bra026666	Bra017451
	452	A06	24666391	24666142	At5g46630	**Bra025009**	Bra022052	Bra017537
	473	A07	8537339	8537527	At1g22340	Bra031388	**Bra012324**	Bra016424
	474	A07	9114152	9114414	At1g20670	**Bra025837**	Bra012243	Bra016456
	566	A08	20712404	20712596	At1g07920	**Bra018669**	Bra031602	Bra030707
	654	A10	1570075	1569913	At1g06080	**Bra015473**	Bra032437	Bra030638
TWO COPY GENES	46	A01	18882651	18882770	At5g52140	**Bra028293**	Bra022579	-
	55	A01	23626196	23626459	At3g16180	**Bra027185**	Bra021168	-
	61	A01	25069244	25069502	At3g02180	-	**Bra021476**	Bra001035
	87	A02	5079784	5080047	At5g20540	-	**Bra006563**	Bra020109
	113	A02	9852697	9852644	At1g66370	Bra004162	**Bra039763**	-
	153	A02	25486260	25486523	At5g23940	Bra009716	-	**Bra029388**
	168	A03	2298612	2298349	At5g12420	-	**Bra006160**	Bra023377
	200	A03	11193078	11192830	At2g47460	Bra004456	-	Bra000453
	234	A03	20936196	20936267	At5g23260	**Bra013028**	Bra026507	Bra029365
	235	A03	20936271	20936494	At5g23260	**Bra013028**	Bra026507	Bra029365
	249	A03	24785451	24785715	At4g22950	-	**Bra019343**	Bra020826
	319	A04	18584148	18584406	At2g45550	Bra004921	**Bra039330**	-
	444	A06	22352521	22352784	At3g27640	**Bra025293**	-	Bra039073
	460	A07	1577014	1576769	At2g18230	**Bra039627**	-	Bra037229
	467	A07	6402416	6402153	At1g29120	-	Bra030121	Bra010851
	490	A07	12392864	12392917	At3g57530	Bra007334	-	**Bra003287**
	536	A08	12108300	12108552	At4g35150	-	Bra017699	**Bra034678**
	545	A08	15271728	15271631	At4g36760	Bra011704	-	**Bra010574**
	596	A09	8214071	8213980	At1g61890	**Bra027073**	Bra028379	-
	597	A09	8214185	8214078	At1g61890	**Bra027073**	Bra028379	-
	604	A09	16501688	16501868	At5g46350	Bra025021	Bra022033	**Bra017561**
	605	A09	16667316	16667053	At5g46040	-	Bra022016	**Bra017582**
	606	A09	16682960	16683223	At5g46040	-	Bra022016	**Bra017582**
	608	A09	19871591	19871427	At1g32780	**Bra023290**	Bra010185	-
	615	A09	24192808	24192545	At1g23380	**Bra024593**	-	Bra016348
	666	A10	8067999	8067852	At5g57655	**Bra002710**	Bra020426	-
	670	A10	8789727	8789464	At5g59340	**Bra002576**	Bra020321	-
ONE COPY GENES	40	A01	14766344	14766081	-	-	-	**Bra029909**
	41	A01	14767003	14766741	-	-	-	**Bra029909**
	129	A02	16530545	16530808	At4g01590	-	-	**Bra008554**
	178	A03	5992774	5992961	-	-	-	**Bra029035**
	223	A03	18448338	18448491	At3g20360	-	-	**Bra001785**
	578	A09	3996947	3996684	At2g11810	-	-	**Bra037199**
	266	A03	29733949	29734212	-	-	**Bra017680**	-
	268	A03	30599723	30599787	At4g36940	-	**Bra017808**	-
	472	A07	7616363	7616100	-	-	**Bra012436**	-
	49	A01	21397784	21397864	At3g19870	-	**Bra038237**	-
	120	A02	12485621	12485884	At1g72110	-	**Bra008008**	-
	128	A02	15935110	15935361	At1g80200	-	**Bra008487**	-
	148	A02	24005490	24005690	-	-	**Bra020642**	-
	285	A04	7140471	7140542	-	**Bra028251**	-	-
	378	A05	20048141	20048392	-	**Bra027271**	-	-
	445	A06	22716443	22716194	At3g26610	**Bra025216**	-	-
	501	A07	16958538	16958801	At1g65590	**Bra004121**	-	-
	513	A07	20340243	20340188	At1g74790	**Bra015893**	-	-
	655	A10	4270200	4270396	At1g02390	**Bra033323**	-	-
	656	A10	4270415	4270484	At1g02390	**Bra033323**	-	-
	657	A10	4410198	4410053	-	**Bra033297**	-	-
	672	A10	9364412	9364675	-	**Bra002467**	-	-
	673	A10	9364744	9365007	-	**Bra002467**	-	-
	677	A10	10858935	10858966	-	**Bra002214**	-	-

^z^ The triplicated chromosome blocks are denoted according to the classification of the *B. rapa* genome annotation databases (BRAD) [25,31]. The triplicated chromosome blocks are classified as one least fractionized block (LF) and two moderately fractionized blocks (MF1, MF2). Genes with *BRAMI-*1 insertion in introns are denoted in bold and their NIPs are denoted in plain letters.

**Figure 4:**
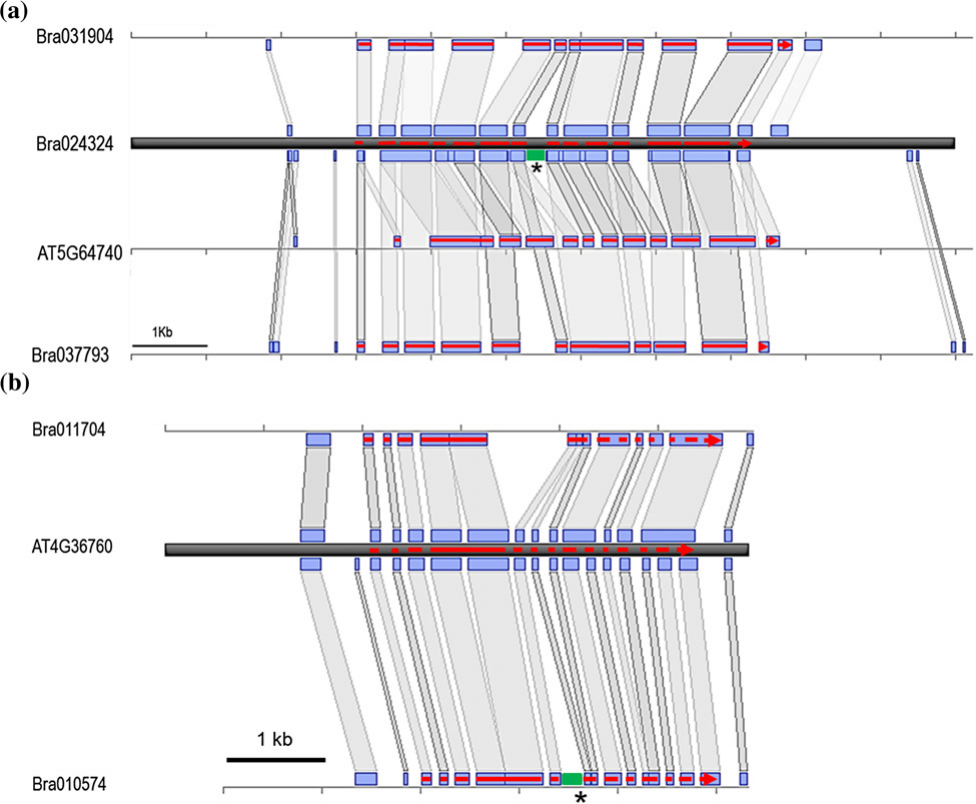
**Microsynteny between the genomic regions with *BRAMI*-1 insertions and homologous blocks in *B. rapa *and *A. thaliana*.** (**a**) Genomic region including 3 kb upstream from the start codon and 3 kb downstream from the stop codon of Bra024324 compared with those of its two paralogs and *Arabidopsis* ortholog. (**b**) Genomic region including 2 kb upstream from the start codon and 0.3 kb downstream from the stop codon of Bra010574 compared with those of its paralog and *Arabidopsis* ortholog. Genomic organization, such as exon and intron location, is based on annotation information in BRAD for *B. rapa* and TAIR for *A. thaliana*. Red lines indicate exons of each gene annotation. The gray bars connecting boxes on genome sequences indicate synteny blocks present in both sequences. The position of the MITE insertion is indicated by both an asterisk and a green block. The map was generated based on nucleotide sequence similarity determined by BLASTn search.

For example, Bra024324 gene was annotated as having 13 exons and included the *BRAMI-*1 insertion in the 7^th^ intron. Its two NIPs (Bra031904, Bra037793) and its *Arabidopsis* ortholog (At5g64740, *CELLULOSE SYNTHASE 6*) have similar structures in which the exonic regions share conserved sequences with Bra024324 (Figure 4a). Another gene, Bra010574, which has the *BRAMI-*1 insertion in 5^th^ intron, showed conserved CDS sequences without any change of gene structure compared to its NIPs (Bra011704) and its *Arabidopsis* ortholog (At4g36760, 15 ORF, *N-1-NAPHTHYLPHTHALAMIC ACID BINDING PROTEIN*) (Figure 4b).

### Transcriptional changes of *B. rapa* genes containing *BRAMI-*1 insertions

**Figure 5:**
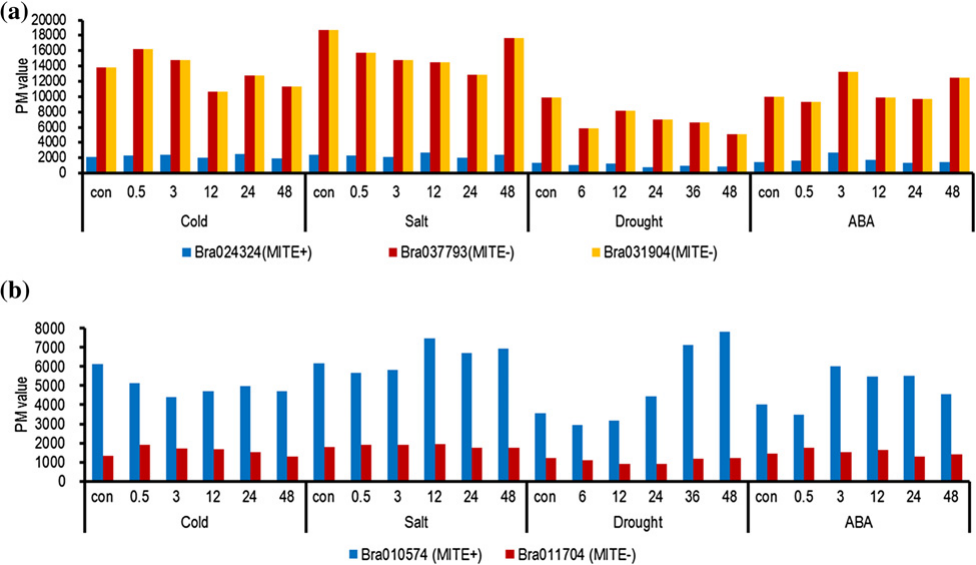
**Comparison of expression profiles between genes with *BRAMI-*1 insertions and their NIPs.** (**a**) Expressions of Bra024324 and its two NIPs, Bra031904 and Bra037793, were analyzed by searching a microarray database of *B. rapa* treated with cold (4°C), salt (250 mM NaCl), drought (air-drying), or ABA (100 μM). (**b**) Expression of Bra010574 and its NIP, Bra011704, were compared. MITE+ and MITE- indicate genes with the *BRAMI-*1 insertion and their NIPs, respectively.

Even though most of the *BRAMI-1* insertions were found in introns or UTRs, some modification of gene expression might still be mediated by *BRAMI-*1. Therefore, we analyzed expression level changes by comparison to NIPs using a *B. rapa* microarray database. Among the 46 multicopy genes with *BRAMI-*1 insertions (20 tri-copy genes and 26 di-copy genes), only six were present along with their NIPs in the microarray database. Of the six genes with *BRAMI-*1 insertions, only Bra039627 showed similar expression to that of its NIPs, regardless of stress treatments. One gene, Bra024324, showed decreased expression and four genes, Bra027185, Bra039330, Bra034678, and Bra010574, showed increased expression compared to that of their NIPs (Figure 5).

The expression of Bra024324, which contains a *BRAMI-*1 insertion, was severely decreased compared to that of its NIPs, Bra031904 and Bra037793, under normal conditions and also under the four stress treatment conditions, indicating that Bra024324 gene expression was maintained at a very low level even though the *BRAMI-*1 insertion did not affect exons (Figure 5a). By contrast, expression of Bra010574, with a *BRAMI*-1 insertion, was more than 3-fold higher than expression of its NIP Bra011704 under control and all four treatment conditions (Figure 5b).

### Survey of MITE insertion polymorphisms (MIPs) and estimation of activation dates

To analyze *BRAMI-*1’s transposition activity and insertion time, we designed 50 MIP primers, 25 for *B. rapa* and 25 for *B. oleracea*, from the flanking regions of the *BRAMI-*1 insertions, especially insertions in genic regions (Additional file 2). The positions of the 25 *B. rapa* MIPs are denoted as arrows on the *in silico* map (Figure 2). Almost all of the primer pairs revealed polymorphisms (48 in 50 pairs; 96%) among seven accessions belonging to three *Brassica* species, indicating that the *BRAMI-*1 members have been continuously activated during diversification of the *Brassica* genome. Moreover, there was high polymorphism within species, with seven (14%), six (12%), and ten (20%) polymorphisms among two accessions of *B. napus,* two accessions of *B. rapa,* and three accessions of *B. oleracea,* respectively.

**Figure 6:**
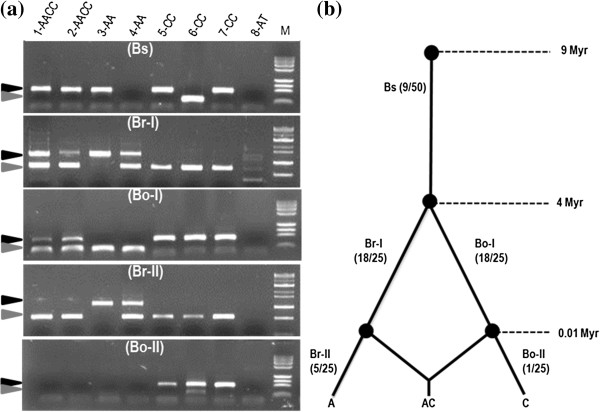
**MITE insertion polymorphism (MIP) analysis and estimation of insertion time.** MIP patterns were classified into 5 groups (Bs, Br- I, II and Bo- I, II), based on existence of MIPs between species. (**a**) Gel electrophoresis of five MIPs (Bo-23, Br-6, Br-3, Bo-10, Bo-21, ordered from the top, for more information on the MIP IDs refer to Additional file 2). The lane numbers (1 to 8) indicate plant materials used, as described in Table 1. A, C, and AC represent the genomes of *B. rapa*, *B, oleracea*, and *B. napus*, respectively. AT indicates *A. thaliana*. M, molecular size marker. The presence or absence of an insertion is denoted by a black or gray arrowhead, respectively. (**b**) Estimated insertion timing for the five MIP groups during the evolution of *Brassica* species [27,36,37].The number within the parentheses indicates the corresponding number of MITE members belonging to the particular group (based on the analysis in panel **a**).

We grouped the 50 MIPs into three different groups: Bs (common to both species), Br (*B. rapa*-unique), and Bo (*B. oleracea*-unique), to deduce the tentative insertion times (Figure 6a). The Br and Bo MIPs were further classified into two subgroups, -I and –II, based on the presence or absence of the insertion in their allopolyploid species *B. napus*. Among the 25 *B. rapa* MIPs, 3, 17, and 5 were Bs, Br-I, and Br-II type insertions, respectively, and among the 25 *B. oleracea* MIPs, 6, 18, and 1 were Bs, Bo-I, and Bo-II types, respectively. Overall, 18% were shared in the *Brassica* genus, and 82% were species-unique insertions (Figure 6b).

Phylogenetic analysis based on the 50 MIP profiles revealed four distinct clusters at the 0.30 genetic similarity coefficient level (Additional file 3). *Arabidopsis* was separated from *Brassica* accessions with a genetic similarity coefficient of 0.16. Three *Brassica* species each formed a distinct cluster with two or three accessions belonging to each species, corresponding well with the phylogeny of *Brassica* species. Each MIP reflects the insertion time at that genomic position and thus MIP-based genotyping and phylogenetic analysis will be a good tool for study of genetic diversity in the *Brassica* genus. We also confirmed that the MIPs are clearly distinguishable on agarose gels, heritable and reproducible, characteristics beneficial as DNA markers. A MIP between two *B. oleracea* accessions, Bo-19, segregated according to a normal Mendelian 1: 2: 1 ratio in a survey of 94 F_2_ progeny of a cross between the two accessions (Additional file 4).

## Discussion

### Structure, distribution and evolution of *BRAMI-*1 in the *B. rapa* genome

*BRAMI-*1 exhibits the basic characteristics of conventional *Stowaway*-like MITEs, which include small size, TIRs, and TSDs, and also possesses a potential DNA hairpin-like secondary structure*. BRAMI-*1 elements have a highly conserved 33 bp TIR region that is rich in A + T nucleotides (>69%) and a 194 bp internal region. In plants, most MITEs are classified as either *Tourist*-like or *Stowaway*-like. *Tourist*-like MITEs are regarded as deletion derivatives of full-length autonomous TEs, such as *mPing* derived from *Pong* and *PIF*[13,32,33]. The origin of *Stowaway*-like MITEs is unclear due to the lack of sufficient sequence similarity to known autonomous TEs [1,34]. However, numerous *Stowaway*-like MITEs can be cross-mobilized by distantly related *Mariner*-like elements (MLEs) to generate high copy numbers [13,35]. However, we could not identify the trans**-**acting autonomous element for the *BRAMI-*1 elements in this study.

### Rapid amplification of *BRAMI-*1 elements in the *Brassica* genus

The genus *Brassica* is an excellent model plant to study polyploidization-mediated genome evolution because allotetraploid species like *B. juncea, B. napus,* and *B. carinata* evolved very recently from the three diploid species *B. rapa, B. oleracea,* and *B. nigra*, and even the diploid *Brassica* species have triplicated genome features that arose approximately 13 million years ago (MYA) [26,27,36]. The estimated copy numbers of the *BRAMI-*1 elements were similar in two closely related *Brassica* species: 1440 and 1464 in *B. rapa* and *B. oleracea*, respectively supporting that *BRAMI-*1 elements were actively amplified in both *Brassica* species [27,36,37]. This is the first MITE found to exhibit very high copy numbers in *Brassica*, although one medium copy number *Brassica Stowaway* MITE, named *Brasto*, was recently characterized [38].

*BRAMI*-1 shares 77% similarity with the *A. thaliana* MITE *ATTIRTA-*1, suggesting that they evolved from a common ancestor of the *Brassica* and *Arabidopsis* lineage. Phylogenetic analysis revealed that *ATTIRTA-*1 and *BRAMI-*1 elements have clearly different evolutionary histories. The *ATTIRTA-*1 elements showed a high amount of variation even though their copy numbers were small compared to those of the *BRAMI-*1 members, indicating that the *ATTIRTA-*1 members were maintained in the *Arabidopsis* genome without further amplification after the split from the *Brassica* lineage 13–17 MYA [27,36]. By contrast, members derived from *B. rapa* (red), *B. oleracea* (blue), and *B. napus* (black) are highly conserved and interrelated with each other, demonstrating that the members were actively amplified in the *Brassica* lineage after divergence from *Arabidopsis* (Figure 3)*.* This is consistent with a report showing highly active TE amplification in *B. oleracea*[28]. We assume that several transpositional bursts may have been responsible for the amplification of the *BRAMI-*1 members in the *Brassica* lineage [16,39,40].

### The putative role of *BRAMI-*1 in *B. rapa* genome evolution

There have been many reports of MITEs involved in the evolution of genes and genomes. MITEs are often inserted in genic regions such as promoter regions, UTRs, introns, or exons and can influence the expression of genes [1,2,16,19,34]. MITE insertion into the various functional regions of a gene can modify its transcriptional activity, cause silencing, and up- or down-regulation of gene expression [34,41]. We found 697 *BRAMI-*1 elements were dispersed across the whole genome (Figure 2). A total of 626 members (90%) were identified in 177 Mb of intergenic spaces and 71 members (10%) were identified in 79 Mb of gene spaces in the 256 Mb *B. rapa* pseudo chromosome sequences. Among the 697 elements, 548 members (78.6%) were located within 3 kb of genic regions and all the 71 copies found in genic regions were resided in introns. The 33 Mb intronic regions exhibited 65% A + T composition, which was much higher than that of 46 Mb exonic regions (54% A + T composition). This insertion target site preference for non-coding sequences of genic regions is similar to the insertion preference of *mPing* in rice, which is more often found in A + T rich non-coding sequence than in G + C rich exonic regions [19].

We showed that *BRAMI*-1 insertion might be one of the causal forces for modification of gene expression. When we compared the expressions of several genes harboring *BRAMI-*1 within their genic regions with those of NIPs, most of the genes with *BRAMI*-1 insertions showed different expression patterns than their NIP counterparts (Figure 5). Comparison of microsynteny between regions with *BRAMI*-1 insertions and their non-insertion homologous genes in *B. rapa* and *A. thaliana* showed relatively conserved coding sequences but more sequence variation in introns and UTRs, including from the *BRAMI*-1 insertions (Figure 4). The observed changes in transcription levels might arise from *BRAMI*-1 insertions into intronic or UTR regions, similar to a recent report showing an enhancing effect of *mPing* near rice genes [19]. Further intensive study of whole transcriptome profiles will be necessary to address MITE effects on gene expression.

### *BRAMI-*1 elements are active up to the present in *Brassica* genera

MIP patterns showing insertions specific to certain species or accessions elucidate the timing of insertion events. Among 50 MIPs, nine (18%) *BRAMI*-1 elements were found in both *B. rapa* and *B. oleracea*, indicating that they were inserted into the regions before *B. rapa* and *B. oleracea* diverged from each other 4 MYA [27,36]. The other 41 (82%) were unique to one species or the other, indicating they were inserted after the divergence of the two lineages. Among the 41 species-specific members, six (8%) showed no insertion in *B. napus* (the allopolyploid product of *B. rapa* and *B. oleracea*) indicating that they inserted into each genome after allopolyplidization 0.01 MYA [36] (Figure 6). Some MIPs were found between accessions of same species, and the MIPs segregated normally in an F_2_ population, opening a new window for MIP-based marker development for marker-assisted selection and other breeding applications in *Brassica* crops. Overall, the MIPs revealed that *BRAMI*-1 elements were gradually inserted into the *Brassica* genome during various events and remained active up to the present.

## Conclusions

We characterized a high copy *Stowaway* family MITE, named as *BRAMI*-1, in three *Brassica* crops and showed its putative role in the evolution of the highly duplicated *Brassica* genome based on comparative genomics analysis. MIP analysis revealed that the *BRAMI*-1 elements were dispersed into whole *Brassica* genome by gradual amplification. We also propose effective utilization of the elements as DNA markers for breeding and evolution of duplicated genes.

## Methods

### Identification and characterization of *BRAMI*-1

We analyzed a repeat-rich *B. rapa* BAC clone sequence, KBrB059A03 (AC189406), to find high copy repeat elements using MUST, a *de novo* program for MITE analysis, with the default parameters [42]. The BAC clone contained 139 kb of highly repetitive sequence. The structure of the TIRs was analyzed using weblogo [43]. The hypothetical DNA hairpin-like structure was predicted using the mfold application [44].

The new MITE was used as a query to retrieve its family members from a local database (http://im-crop.snu.ac.kr/) that includes 256 Mb of 10 pseudo-chromosome sequences from *B. rapa,* 425 Mb of *B. oleracea* shotgun sequences, 15 Mb of *B. napus* shotgun sequences, and the whole genome sequence of *A. thaliana,* using the approach suggested by Wicker et al. (2007) [10]*.* BLASTn with default parameters [45] and a threshold E-value of 1E^-10^ was employed to search for MITE family members. The insertion sites of 697 elements and their flanking regions were annotated using the *B. rapa* genome database [31].

### Estimation of copy number

The copy number of *BRAMI*-1 in the *B. rapa* genome (529 Mb) was estimated from the number of copies identified in 256 Mb of 10 pseudo-chromosome sequences from *B. rapa*[25]. The copy numbers in the *B. oleracea* and *B. napus* genomes were estimated by considering the hit numbers in the available genome shotgun sequences. A total of 425 Mb of *B. oleracea* sequences derived from 680,894 genome shotgun sequences with an average length of 700 bp [46] and 15 Mb of *B. napus* shotgun sequences derived from 52,099 genome shotgun sequences (GSS) with an average length of 700 bp were downloaded from GenBank (NCBI) and used as local databases. The copy numbers of *BRAMI*-1 in *B. oleracea* and *B. napus* were estimated using the previously reported formula [28]: [(1/genome coverage)/2] x number of hits {[1 + [(average GSS) -TIR length x2)/(average GSS length + TIR length x2)]}. Relatively intact copies with more than 80% coverage of the *BRAMI*-1 structure were collected from the three *Brassica* species for phylogenetic analysis. Multiple sequence alignment was conducted using ClustalW and phylogenetic analysis was performed based on the neighbor joining method in MEGA5 [47]. In *A. thaliana*, *ATTIRTA*-1 was the most closely related element to *BRAMI*-1*,* so it was included in the phylogenetic analysis. Tree topologies were evaluated using bootstrap analysis with 500 replicates for the neighbor-joining method [47].

### Expression analysis of *B. rapa* genes with *BRAM1*-1 insertions

We investigated expression modification of genes that had a MITE insertion inside of the gene structure by comparison with their syntenic paralogs using a 24 K microarray database (http://nabic.rda.go.kr) [48]. The microarray database represented ca. 24,000 unigenes generated from cDNA libraries of *B. rapa* ssp. *pekinensis* (inbred line ‘Chiifu’) and provided transcriptome profiling of changes induced by abiotic stress treatment. A given probe sequence and its ID in the microarray were searched using the coding sequence of the gene as a query. The perfect match (PM) values of probes were retrieved and processed to identify expression patterns, as described previously [48].

### **MITE Insertion polymorphism (MIP**)

To inspect insertion polymorphisms and thus infer activation times, we used seven *Brassica* accessions belonging to three species and *A. thaliana* ecotype Columbia (Table 4). DNA was extracted from fresh leaf samples using the CTAB method [49]. In addition, a total of 94 F_2_ progeny from a cross between *B. oleracea* accessions C1234 and C1184 were used for segregation pattern analysis of MIPs.

**Table 4:** Plant materials used for MIP analysis

	**Genome**	**Species**	**Accessions (cultivars)**
1	AACC	*B. napus*	Tapidor
2	AACC	*B. napus*	Ningyou7
3	AA	*B. rapa*	Chiifu
4	AA	*B. rapa*	Kenshin
5	CC	*B. oleracea*	C1234
6	CC	*B. oleracea*	C1184
7	CC	*B. oleracea*	C1235
8	AT	*A. thaliana*	Columbia

We designed 50 primer pairs, 25 using shotgun sequences of *B. oleracea* (Bo 1–25) and 25 using the *B. rapa* pseudo-chromosome sequences (Br 1–25), from the flanking sequences of *BRAM1*-1 insertion sites using the Primer3 software program [50] (Additional file 2). PCR was conducted in 20 μL total volume containing 10 ng DNA, 10 pmol each primer, 250 μM dNTPs, and 1 unit *Taq* DNA polymerase (VIVAGEN, Republic of Korea). PCR conditions were as follows: 5 min at 94°C, 38 cycles of 95°C for 30 sec, 56°C-62°C for 30 sec, and 72°C for 60 sec, with a final extension at 72°C for 5 min, using a MG96G thermo cycler (LongGene Scientific Instruments, China). PCR products were analyzed using 1% agarose gel electrophoresis and visualized on a UV trans-illuminator after ethidium bromide staining.

For MIP marker analysis, each band was scored as ‘1’ or ‘0’ for presence or absence, respectively. Jaccard’s similarity coefficient and a dendrogram of the genetic relationship according to Unweighted Pair Group Method with Arithmetic Average (UPGMA) analysis were determined by the NTSYS-pc program (Numerical Taxonomy & Multivariate Analysis System) [51].

## Competing interests

The authors declare that they have no competing interests.

## Authors’ contribution

PS and TJY initiate the research. PS, SCL, JL, and NKI carried out the molecular experiments, interpreted the results. PS and BSC performed the bioinformatics analyses. MJ and BSP provided suggestion for the manuscript preparation and writing. PS and TJY wrote the manuscript. All authors critically read and approved the final version of the manuscript.

## Supplementary Material

Additional file 1 **Physical position of the 697 members in the *B. rapa *genome.** Click here for file

Additional file 2 List of Primers used for MITE insertion polymorphism analysis. Click here for file

Additional file 3 **Phylogenetic analysis of MIPs.** Dendrogram based on Jaccard’s similarity coefficient of 50 MIPs among eight genotypes constructed using the UPGMA method. Click here for file

Additional file 4 **MIP survey of 94 *B. oleracea *F**_**2**_** plants from a cross between parental lines C1234 (P1) and C1184 (P2).** The primer MIP-M1-19 was used and a ratio of 22:50:21 was observed for genotypes P1: P1/P2 : P2. Click here for file
