# Supplementary figures and images for: Characterization of a new high copy Stowaway family MITE, BRAMI-1 in Brassica genome
Source: BMC Plant Biol. 2013 Apr 2;13:56. doi: 10.1186/1471-2229-13-56 (PMC3626606; doi:10.1186/1471-2229-13-56)

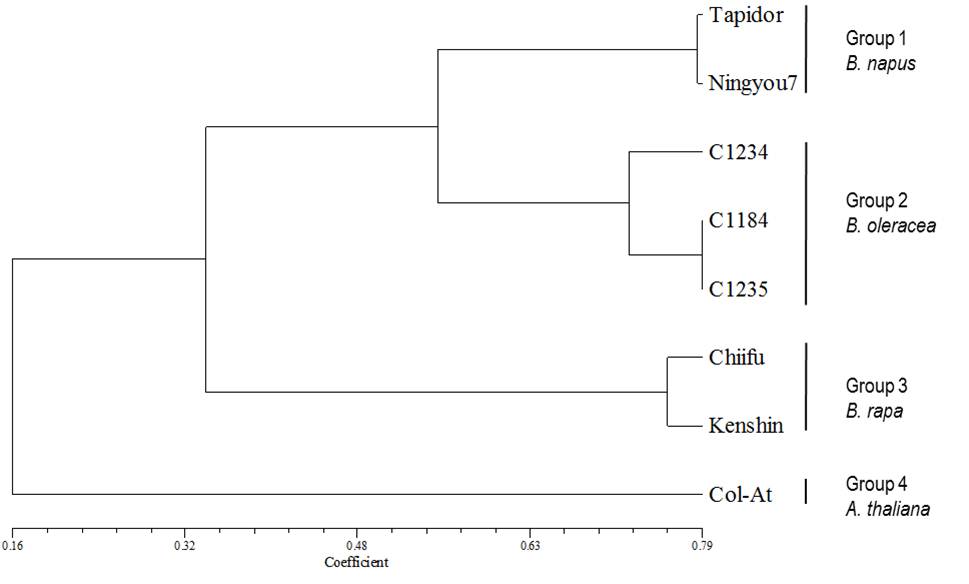

Supplement: Additional file 3 — Phylogenetic analysis of MIPs. Dendrogram based on Jaccard’s similarity coefficient of 50 MIPs among eight genotypes constructed using the UPGMA method. [file 1471-2229-13-56-S3.jpeg]
